# Simultaneous depletion of RB, RBL1 and RBL2 affects endoderm differentiation of human embryonic stem cells

**DOI:** 10.1371/journal.pone.0269122

**Published:** 2022-11-22

**Authors:** Shota Nakanoh, Juned Kadiwala, Laetitia Pinte, Carola Maria Morell, An-Sofie Lenaerts, Ludovic Vallier

**Affiliations:** 1 Division of Embryology, National Institute for Basic Biology, Okazaki, Aichi, Japan; 2 Wellcome Trust–MRC Cambridge Stem Cell Institute, University of Cambridge, Cambridge, United Kingdom; 3 Department of Surgery, University of Cambridge, Cambridge, United Kingdom; 4 National Institute for Health and Care Research Cambridge Biomedical Research Centre Human Induced Pluripotent Stem Cells Core Facility, University of Cambridge, Cambridge, United Kingdom; 5 Wellcome Sanger Institute, Wellcome Genome Campus, Hinxton, United Kingdom; Macau University of Science and Technology, MACAO

## Abstract

RB is a well-known cell cycle regulator controlling the G1 checkpoint. Previous reports have suggested that it can influence cell fate decisions not only by regulating cell proliferation and survival but also by interacting with transcription factors and epigenetic modifiers. However, the functional redundancy of RB family proteins (RB, RBL1 and RBL2) renders it difficult to investigate their roles during early development, especially in human. Here, we address this problem by generating human embryonic stem cells lacking RB family proteins. To achieve this goal, we first introduced frameshift mutations in *RBL1* and *RBL2* genes using the CRISPR/Cas9 technology, and then integrated the shRNA-expression cassette to knockdown RB upon tetracycline treatment. The resulting RBL1/2_dKO+RB_iKD cells remain pluripotent and efficiently differentiate into the primary germ layers *in vitro* even in the absence of the RB family proteins. In contrast, we observed that subsequent differentiation into foregut endoderm was impaired without the expression of RB, RBL1 and RBL2. Thus, it is suggested that RB proteins are dispensable for the maintenance and acquisition of cell identities during early development, but they are essential to generate advanced derivatives after the formation of primary germ layers. These results also indicate that our RBL1/2_dKO+RB_iKD cell lines are useful to depict the detailed molecular roles of RB family proteins in the maintenance and generation of various cell types accessible from human pluripotent stem cells.

## Introduction

During cell cycle progression, stem cells are subjected to the profound changes which can affect their cell fate decisions. Accordingly, molecular interplays between cell cycle and differentiation have been extensively studied in human and mouse embryonic stem cells (hESCs and mESCs, respectively) [1–3]. For example, the links between the G1 length and the loss of pluripotency have been reported in these cells, implicating that the G1 phase may function as a time window critical for their fate decisions [4–6]. The mechanisms behind these phenomena involve cell cycle regulators directing the activity of key signaling pathways. It was established in hESCs that cyclin-dependent kinase 4 and 6 (CDK4/6) control the activity of Activin/Nodal signaling by modulating the nuclear localization of SMAD2/3 [7]. Nonetheless, contributions of other cell cycle machineries to the cell state transitions remain to be fully unrevealed.

Retinoblastoma tumor suppressor (RB) is an important cell cycle regulator which controls the G1/S transition by binding to E2F transcription factors and blocking the transactivation of genes required for S-phase entry until being phosphorylated by CDK4/6 [8]. *RB* was initially identified as the gene whose inactivation results in human retinoblastoma [9]. Although the role of RB to suppress various tumorigenesis is widely observed over species [10], human retinoblasts are specifically susceptible to mutations in *RB*, suggesting potential species diversity in the molecular traits of this gene [11, 12]. RB is known to be critical for proper organogenesis as mouse embryos lacking functional RB die by embryonic day 15.5 exhibiting defects in hematopoietic, neural and placental development [11, 13–15]. In addition to cellular proliferation and survival, RB also regulates the activities of master regulators of differentiation programs, such as CBFA1 in osteogenesis and MYOD in myogenesis [16, 17]. These studies suggest the vital roles of RB in organogenesis, while its contribution to early development, especially in human embryos, is still unclear. This ambiguity could be due to the functional compensation between RBL1 and RBL2 (also known as p107 and p130, respectively), which together with RB constitute the pocket protein family [12]. Thus, these homologous proteins could mask their roles in early development. In fact, the combined disruptions of RB and RBL1 confer embryonic lethality earlier than single knockout of RB [18], and mouse ESCs devoid of all the RB family proteins exhibit severely restricted differentiation capacity when grafted in immunocompromised mice [19].

Here, we aimed to demonstrate the involvement of the RB family in human early development using hESCs genetically modified to simultaneously impair all the family proteins. To this goal, RBL1 and RBL2 were first disrupted by CRISPR/Cas9-mediated non-sense mutations, and then an inducible knockdown cassette was inserted into a genomic safe harbor to conditionally reduce RB. The resulting RBL1/2_dKO+RB_iKD hESCs were able to proliferate and differentiate normally, but the expression of RB family proteins became critical in the later stage of endoderm differentiation. Taken together, these data suggest that RB proteins are not necessary for cell cycle regulation or for differentiation in early human development. Our results also demonstrate the interest of RBL1/2_dKO+RB_iKD hESCs to dissect the molecular functions of the RB family proteins in a variety of pluripotent and differentiated cells.

## Results

### RB, RBL1 and RBL2 are differentially expressed in pluripotent and differentiating hESCs

**Fig 1:**
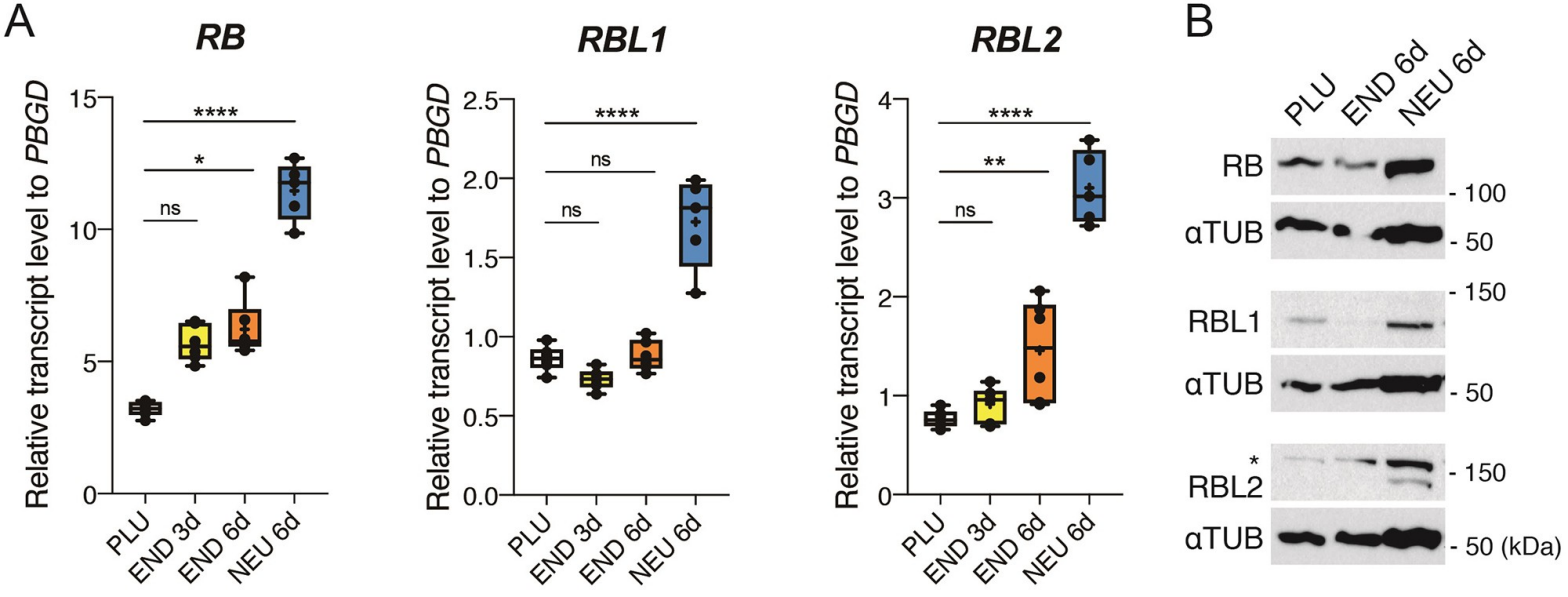
Gene expression analyses of the RB family in the wild type H9 hESCs during directed *in vitro* differentiation. A, Transcript levels measured by qRT-PCR. Box-plot elements: centre line is median; box limits are upper and lower quartiles; whiskers are minimum and maximum; cross is mean. n = 5 or 6 from two or three different experiments. NS: p-value ≥ 0.05, *: p-value < 0.05, **: p-value < 0.01, ****: p-value < 0.0001 (Ordinary one-way ANOVA and Kruskal-Wallis test were used based on Shapiro-Wilk normality test). B, Western blot analysis of RB, RBL1 and RBL2 proteins. α-TUBLIN was used as a loading control. Star indicates non-specific bands stained with the RBL2 antibody.

We first examined the expression levels of RB, RBL1 and RBL2 in pluripotent and differentiating hESCs. Quantitative polymerase chain reaction (qPCR) analyses revealed the gradual increase of their transcripts during differentiation into definitive endoderm and neuroectoderm (Fig 1A). *RB* and *RBL2* showed a limited increase during endoderm differentiation, while all the *RB* genes were induced during neuroectoderm differentiation. Consistent with the severe phenotype of RB-deficient mice [12], *RB* was more abundantly transcribed than *RBL1* and *RBL2* in general. Western blot analyses confirmed these results at the protein level (Fig 1B). Altogether, our data suggest that RB family genes are expressed in pluripotent hESCs, and that their expression levels tend to increase during differentiations, especially toward the neuroectoderm lineage.

### Establishment of RBL1/2_dKO+RB_iKD hESC lines

**Fig 2:**
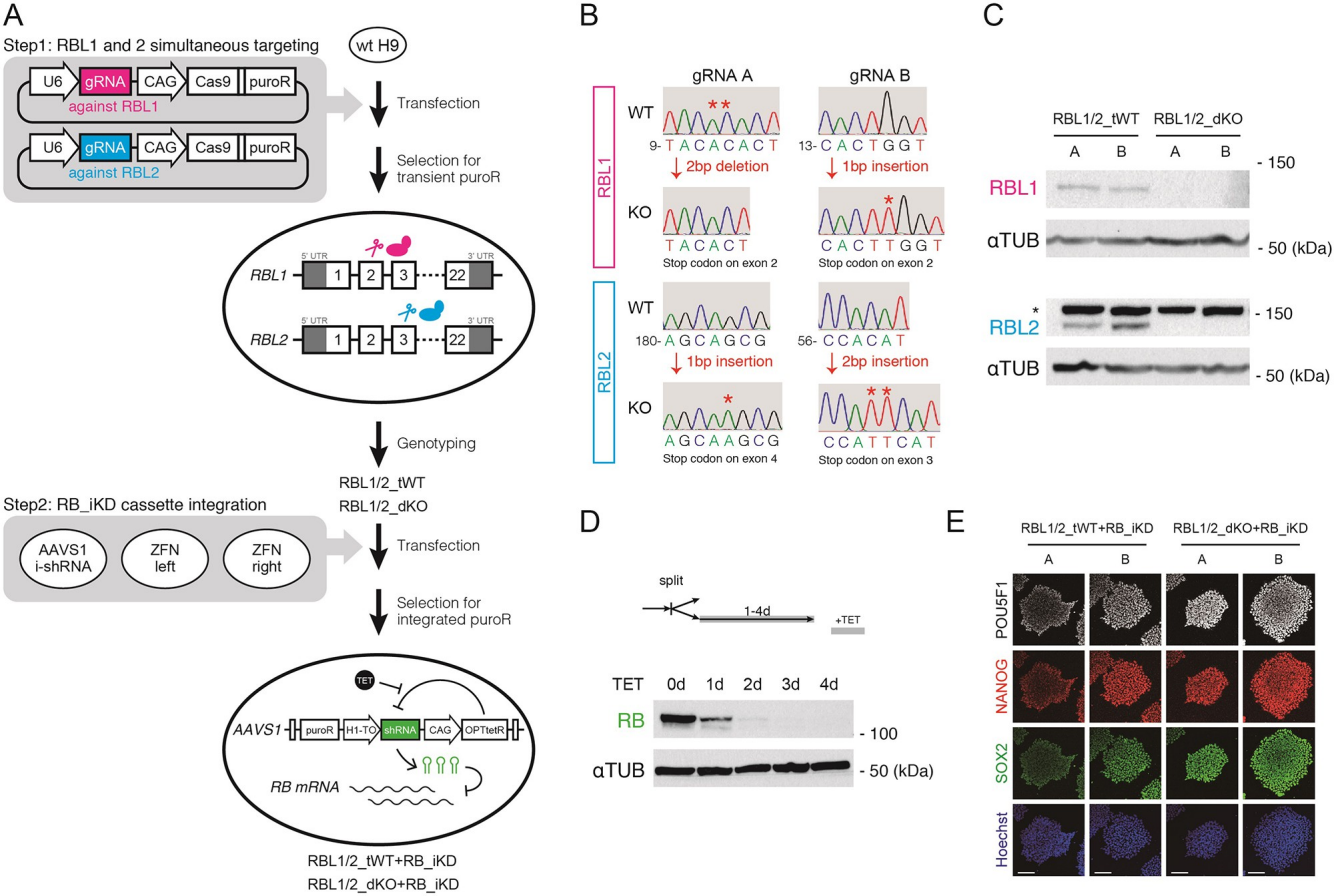
Establishing the platform to disturb all the RB family members. A, The outlined process to generate RBL1/2_dKO+RB_iKD H9 hESCs. First, wild type H9 hESCs were transfected with the all-in-one Cas9 vectors to target *RBL1* and *RBL2* simultaneously. Two combinations of different gRNAs were used as shown as A and B. Following the selection for the transient puromycin resistance and the genotyping assays, RBL1/2_TG cells were then transfected with OPTiKD vectors coding shRNA against RB for genomic integration. Once established, these cells are ready for the RB knockdown inducible by tetracycline administration. B, Genotyping assay by Sanger sequencing of tWT and dKO hESCs from the Step1 in A. Regions containing the indel mutations from the targeting are shown. Numbers indicate the locations in exon 2 and exon 3 of *RBL1* and *RBL2*, respectively. Starred bases are missing in the other genotype. C and D, Western blot analyses of the derived cell lines. C highlights the lack of RBL1 and RBL2 proteins corresponding to the genotypes, and D shows gradual removal of RB protein upon tetracycline treatment in RBL1/2_dKO+RB_iKD hESCs from the gRNA set B. α-TUBLIN was used as a loading control. Star indicates a non-specific band stained with the anti-RBL2 antibody. E, Immunofluorescent images of the hESCs maintained in the pluripotent stem cell condition.

In order to study the function of RB family proteins in human pluripotency and early differentiation, we decided to generate H9 hESC lines lacking all the RB family members (Fig 2A, also see Materials and Methods). First, we simultaneously targeted *RBL1* and *RBL2* using CRISPR/Cas9 (Fig 2A, Step1). Plasmids carrying gRNA- and Cas9-coding sequences were transfected into wild type H9 hESCs, and individual sublines were genotyped after the selection for transient puromycin resistance. Of note, we used two different sets of gRNAs against *RBL1* and *RBL2*, which are referred to as A and B (Fig 2B). Cells carrying nonsense mutations on both alleles of *RBL1* and *RBL2* were maintained as RBL1/2_dKO hESCs, while the ones that retained intact *RBL1* and *RBL2* were kept as control RBL1/2_tWT hESCs (Fig 2B). Western blot analyses showed the absence of the corresponding proteins in RBL1/2_dKO hESCs, thereby confirming that RBL1 and RBL2 were successfully deleted by our gene editing strategy (Fig 2C). Following the generation of RBL1/2_dKO hESCs, we integrated an inducible knockdown cassette into RBL1/2-targeted hESCs based on the OPTiKD platform [20] (Fig 2A, Step2). The resulting RBL1/2_dKO+RB_iKD hESCs and RBL1/2_tWT+RB_iKD hESCs were then grown in the presence of tetracycline to induce the expression of shRNA directed against RB. Western blot analyses showed efficient decrease in RB protein to the undetectable level within three days after induction (Fig 2D). Note that immunocytochemistry confirmed that the resulting hESC lines remain pluripotent in the maintenance condition (Fig 2E). Taken together, these results confirm that our strategy allowed the generation of hESC which can be induced to lack all the RB family proteins. Hereafter, results of RBL1/2_tWT+RB_iKD and RBL1/2_dKO+RB_iKD hESCs of gRNA B are mainly shown and referred to as tWT and dKO hESCs, respectively.

### Depletion of RB, RBL1 and RBL2 affects cell cycle but not pluripotency gene expression

**Fig 3:**
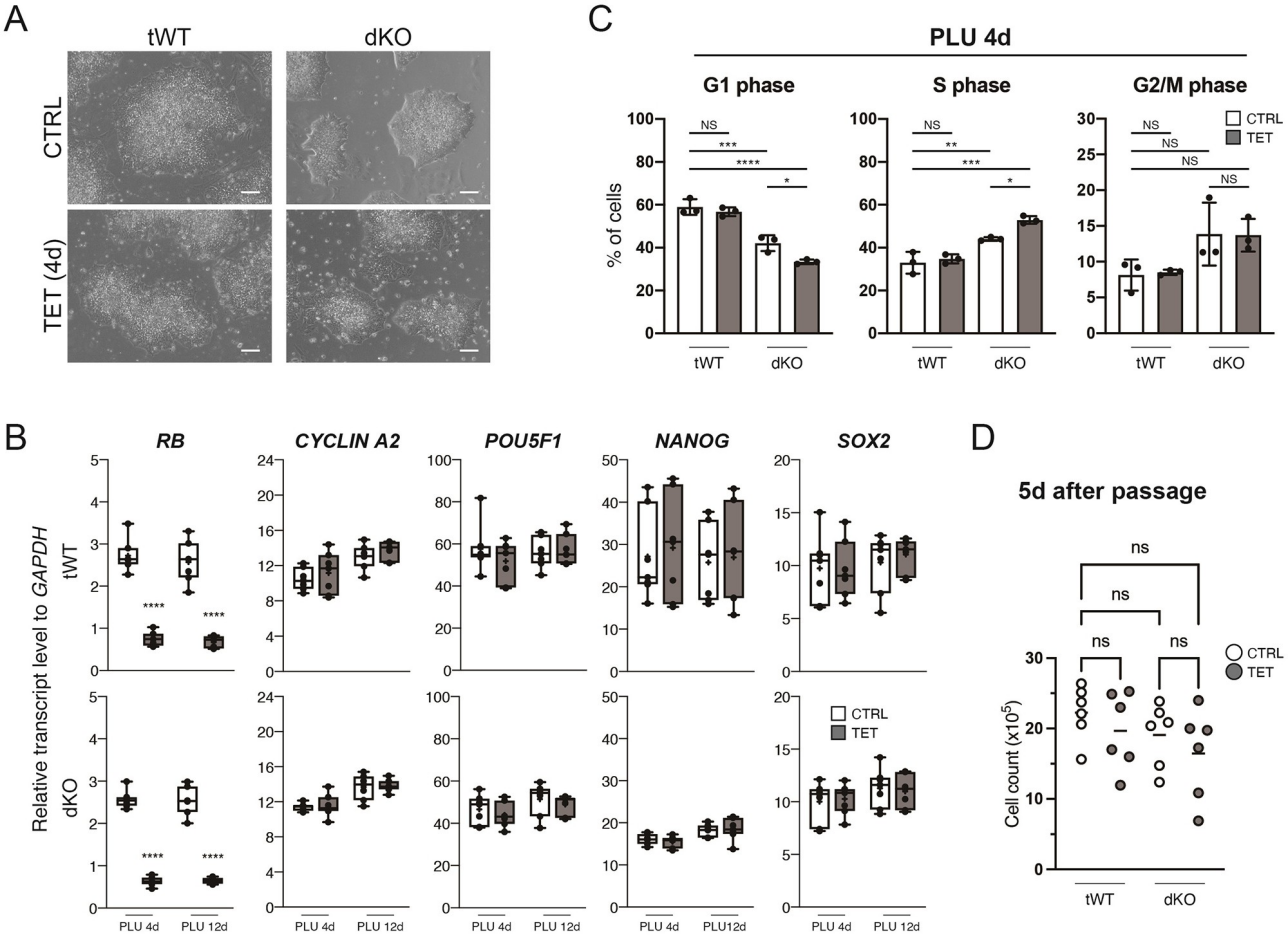
Analyses of the established tWT and dKO hESCs in the pluripotent stem cell culture condition. A, Bright field images of tWT and dKO cells with or without tetracycline. Scale bars represent 100 μm. B, Transcript levels of representative genes in the pluripotent tWT and dKO cells measured by qRT-PCR. Box-plot elements are same as described in Fig 1. n = 7 from three different experiments. To determine statistical significance between CTRL and TET sample groups on the same time point, Student’s t-test and Mann-Whitney test were performed based on Shapiro-Wilk normality test. No mark: p-value ≥ 0.05, ****: p-value < 0.0001. C, Cell cycle profiling of tWT and dKO cells treated with tetracycline for four days. NS: p-value ≥ 0.05, *: p-value < 0.05, **: p-value < 0.01, ***: p-value < 0.001, ****: p-value < 0.0001 (Ordinary one-way ANOVA and Kruskal-Wallis test were used based on Shapiro-Wilk normality test). n = 3. D, Cell counts 5 days after passages. 5.0x10^4^ cells were seeded as single cells and treated immediately with tetracycline. Bars represent means from six samples. Ordinary one-way ANOVA test were used.

Using tWT and dKO hESCs, we examined the impact of the absence of the RB family proteins on human pluripotency. Knockdown of RB induced by the addition of tetracycline did not introduce evident morphological changes in the tWT or dKO cells (Fig 3A) despite the efficient reduction in the *RB* transcripts (Fig 3B and S1 Fig). In addition, the downstream target of the RB-E2F pathway [21], *CYCLIN A2*, and the pluripotency markers, *POU5F1*, *NANOG* and *SOX2* were not affected by the knockdown even in the RBL1/2 double knockout hESCs. We also analysed the cell cycle profiles of the tWT and dKO cells (Fig 3C). Interestingly, absence of RBL1/2 shortened the G1 phase and lengthened the S phase. This change in the cell cycle profile was accentuated by the knockdown of RB. Of note, reduction of RB was not sufficient to alter the cell cycle in tWT hESCs, and G2/M phases were not significantly affected by either of the knockdown or the knockout. We also evaluated proliferation of the tWT and dKO hESCs after 5 days of knockdown and found no significant change in the cell numbers in the absence of the RB proteins (Fig 3D). Taken together, these results suggest that RB genes regulate the cell cycle in pluripotent hESCs, but are dispensable for the maintenance of pluripotency genes.

### Depletion of RB, RBL1 and RBL2 affects late endoderm phase but not cell cycle

**Fig 4:**
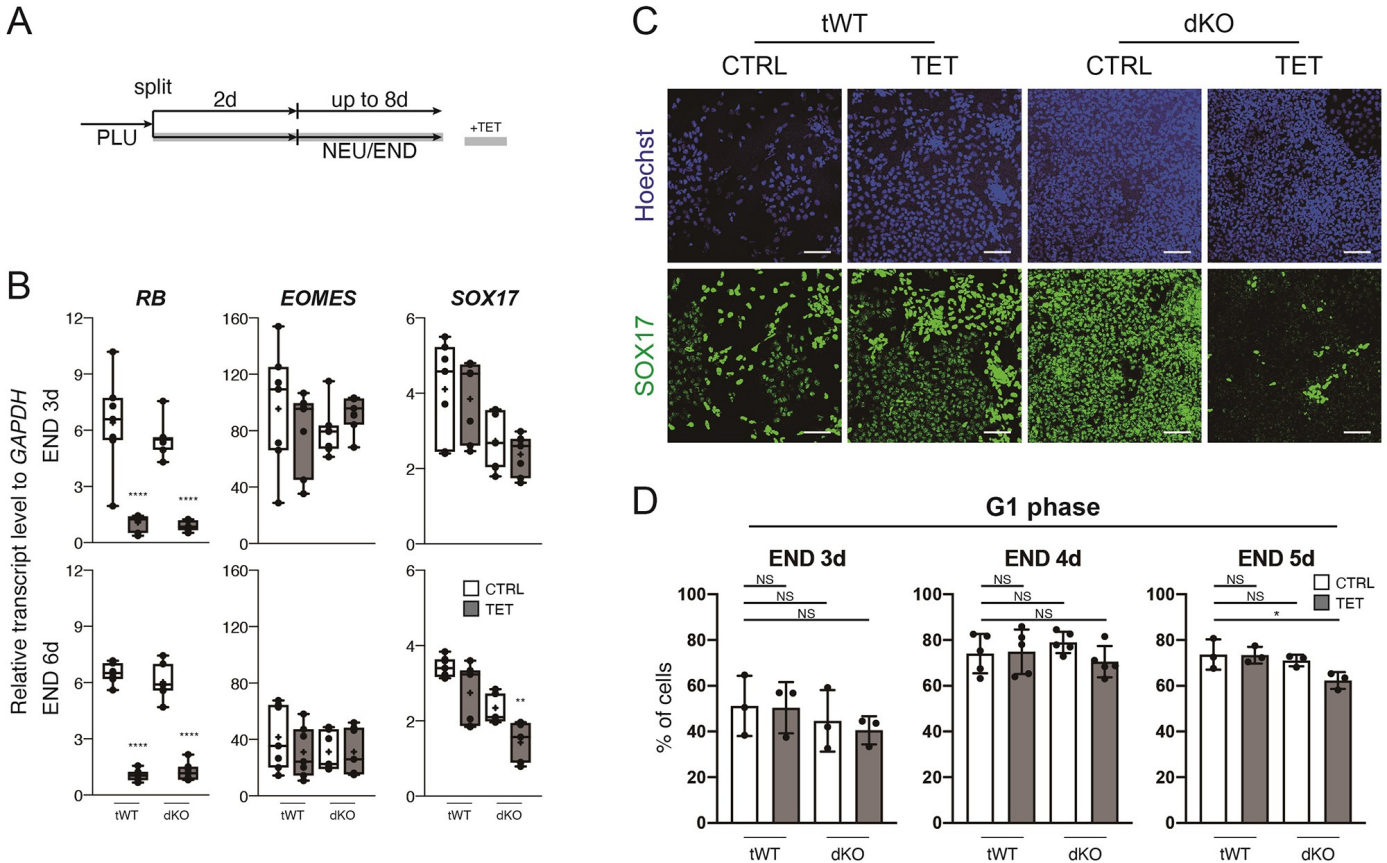
Endoderm differentiation of tWT and dKO hESCs. A, A schematic drawing of the endoderm differentiation. B, Relative expression levels of representative genes during the endoderm differentiation. Box-plot elements are described in Fig 1. n = 7 from three different experiments. To determine statistical significance between CTRL and TET sample groups on the same time point, Student’s t-test and Mann-Whitney test were performed based on Shapiro-Wilk normality test. No mark: p-value ≥ 0.05, *: p-value < 0.05, **: p-value < 0.01, ***: p-value < 0.001, ****: p-value < 0.0001. C, Immunofluorescent images of tWT and dKO cells on endoderm differentiation day 8. Scale bars represent 100 μm. D, Cell cycle profiling of the tWT and dKO cells during the endoderm differentiation. S and G2/M phases are shown in supplemental figures. NS: p-value ≥ 0.05, *: p-value < 0.05 (Ordinary one-way ANOVA and Kruskal-Wallis test were used after Shapiro-Wilk normality test). n = 3 or 5.

In order to study the roles of RB proteins in cell fate decisions, tWT and dKO hESCs were cultured in the conditions to induce endoderm and neuroectoderm differentiation (Fig 4A). We first confirmed that the reduction of *RB* transcripts by the tetracycline treatment was well maintained during differentiation (Fig 4B and S2A Fig). The absence of RB proteins was not associated with either morphological differences or change in neuronal marker (S2A Fig). Thus, we concluded that the roles of RB proteins are limited in the early stage of neuroectoderm induction *in vitro*. We then examined the expression of endoderm markers in the definitive endoderm and foregut endoderm formed on day 3 and 6, respectively (Fig 4B). While the early endoderm marker *EOMES* was largely unaffected at the examined time points, the late endoderm marker *SOX17* was reduced at the foregut stage in dKO hESCs with RB knockdown (Fig 4B). The reduced SOX17 expression was confirmed at the protein level by immunostaining (Fig 4C), and the downregulation of *SOX17* was also observed in the other RBL1/2_dKO+RB_iKD line (S2B Fig). We speculated that this defect in endoderm differentiation might be caused by cell cycle dysregulation. To examine this hypothesis, we analysed the cell cycle profile of tWT and dKO hESCs during differentiation (Fig 4D and S2C Fig). However, the absence of RB family had very little effect on the cell cycle profile. In conclusion, the RB family proteins are necessary in late endoderm differentiation independently of their functions in the cell cycle.

### Depletion of RB, RBL1 and RBL2 confers mesodermal and neuronal expression profiles in the endoderm-induction condition

**Fig 5:**
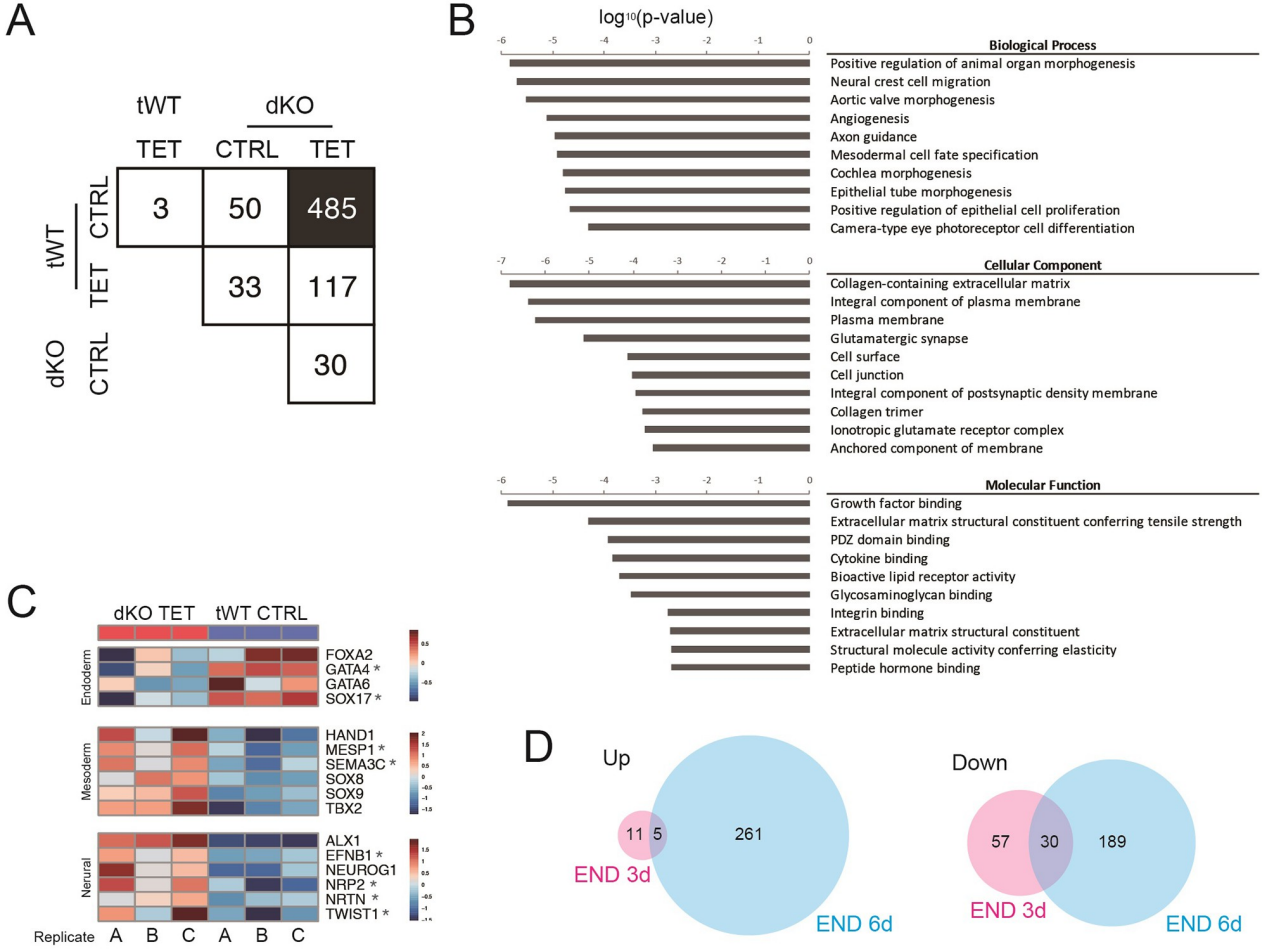
RNA-seq analyses of tWT and dKO cells in endoderm differentiation. A, Number of DEGs identified in each comparison (FC > 1). 349 among 485 DEGs identified in tWT CTRL vs dKO TET were unique in this comparison. B, Gene ontology enrichment analysis on the DEGs detected between tWT CTRL and dKO TET on endoderm day 6 (black box in A). C, Heatmap graphs showing the fold change in the representative gene expression between tWT CTRL and dKO TET cells on endoderm day6. Starred genes were found uniquely in this comparison. D, Genes upregulated or downregulated in dKO TET over tWT CTRL were compared between endoderm day 3 and day6. Limited number of genes were found in both timepoints.

In order to obtain deeper understanding of how RB family proteins are involved in late endoderm differentiation, we performed genome-wide gene expression analysis on tWT and dKO hESCs differentiating into definitive endoderm and foregut using RNA-seq. Depletion of the RB family had only moderate effects on the transcriptome of the definitive endoderm cells in three days of differentiation as comparison between tWT CTRL and dKO TET cells displayed only 103 differentially expressed genes (DEGs) (S3A Fig). Of note, gene ontology enrichment analysis revealed that these DEGs are involved in neural formation, such as eye morphogenesis and telencephalon regionalization, or are cell surface molecules, such as receptors and membrane proteins (S3B Fig). In contrast, absence of RB family proteins had a broader impact on foregut cells, highlighted by the large difference observed between tWT CTRL and dKO TET cells (Fig 5A, filled box: 485 DEGs). Tetracycline treatment itself did not have a great effect (Fig 5A: 3 and 30 DEGs in tWT CTRL vs TET and dKO CTRL vs TET, respectively). The comparison between tWT and dKO CTRLs provided 50 DEGs, while the number decreased to 33 in tWT TET vs dKO CTRL. Similar tendency was seen between tWT CTRL/TET vs dKO TET (the numbers of DEGs were reduced from 485 to 117 by treating tWT with tetracycline). Gene ontology analysis on the 485 DEGs detected between tWT CTRL and dKO TET highlighted biological processes involved in mesoderm and ectoderm, such as neural crest migration, angiogenesis and axon guidance (Fig 5B). Thus, hESCs lacking all the RB proteins seems to follow alternative paths of differentiation. To support this hypothesis, we also analysed the expression of genes representative for three germ layers (Fig 5C). In the dKO TET cells, upregulation of important endoderm markers represented by *FOXA2*, *GATA4*, *GATA6* and *SOX17*, was blocked, while mesoderm and neural markers, such as *HAND1*, *MESP1*, *SEMA3C*, *SOX8*, *SOX9*, *TBX2*, *ALX1*, *EFNB1*, *NEUROG1*, *NRP2*, *NRTN* and *TWIST1*, increased compared with the tWT CTRL cells. Note that most of the 485 DEGs were found not significant in the comparison between dKO TET and tWT CTRL on the day 3 of endoderm differentiation (Fig 5D). Altogether, our data suggest that the absence of RB, RBL1 and RBL2 interferes with late endoderm differentiation while promoting neural and mesodermal differentiation.

## Discussion

There is growing evidence that cell cycle machineries control pluripotency in human pluripotent stem cells [7, 22, 23]. RB and its relative proteins, RBL1 and RBL2, are well known to restrict G1/S transition, and are essential for organogenesis through the regulation of cell cycle and transcription [15]. However, functional redundancy of RB family proteins hampers detailed studies and their involvement in cell fate decisions during human early embryogenesis are yet to be fully understood. To address these issues, we established hESCs carrying non-sense mutations on *RBL1* and *RBL2* together with the inducible knockdown cassette of *RB* (Fig 2). Importantly, these RBL1/2_dKO+RB_iKD cells can be maintained in pluripotent stem cell culture and differentiated efficiently in the differentiation conditions (Figs 2–4), providing the opportunity to compare the effects of depletion of RB, RBL1/2 and all the three proteins in various types of pluripotent and differentiated human cells.

We observed that RB family components were expressed in the pluripotent hESCs and that their expression levels increased moderately toward endoderm lineage (Fig 1). Using the RBL1/2_dKO+RB_iKD hESCs, we examined the influences of RB family proteins on cell cycle structures in the pluripotent and definitive/foregut endoderm cells (Figs 3 and 4). In pluripotency, although the removal of RB did not show significant effects by itself, shorter G1 phase and longer S phase were observed in the pluripotent cells deficient for RBL1 and RBL2, and this tendency was further reinforced by the RB knockdown (Fig 3C). In contrast, cycle profiles were not largely affected by the total absence of the RB family proteins until at least the formation of foregut (Fig 4D). RB is constitutively hyperphosphorylated and thus inactive in mESCs [24, 25], while hESCs have periodic activation of RB as generally found in somatic cells [26–28]. *In vivo* studies showed that mouse embryos lose and regain RB expression at the 4-cell stage and at the late blastocyst stage, respectively [29] and that cell cycle accelerates at the onset of gastrulation [30, 31]. Our results combined with these previous studies suggest that human embryos could gain G1/S restriction point imposed by the RB family proteins by the time of implantation, but then remove it to quicken the cell divisions in forming the primary germ layers during the gastrulation.

The RBL1/2_dKO+RB_iKD hESCs also provided us with insights into the effects of RB proteins on the maintenance and acquisition of cell identity. Even in the total absence of RB, RBL1 and RBL2, hESCs retained the expression of pluripotency markers in the maintenance media (Fig 3B and S1A Fig), early neural markers (S2A Fig), and endoderm markers until the definitive endoderm stages (Figs 4B and 5D, S2B and S3A Figs). These results are consistent with [28], in which the authors observed that the expression levels of pluripotency and early lineage markers were unchanged in the hESCs expressing SV40 T antigen that interferes RB proteins. However, we found that when the differentiation continues toward foregut stage without RB proteins, critical endoderm markers, such as *FOXA2*, *GATA4*, *GATA6* and *SOX17*, were downregulated, and instead mesoderm and neural genes were upregulated (Figs 4B and 4D and 5C). The molecular mechanisms underlying this phenomenon is still unclear, but the unchanged cycle profiles (Fig 4D) seem to exclude the possibility that this deleterious effect of disturbed RB proteins at the gut formation was produced by cell cycle. As previously suggested [16, 17], RB might physically interact and regulate transcription factors to execute differentiation programs in our context. The gene ontology analyses indicated changes in the cell surface compartment, which includes receptor, plasma membrane and extracellular matrix on both endoderm day 3 and 6 (Fig 5B and S3B Fig), suggesting an interesting possibility that RB proteins affect cellular identities through changes in cell surface components. Taken together, these results underline the importance of cell cycle regulators in controlling development beyond their simple roles in cell cycle. Further examinations are awaited.

## Materials and methods

### Maintenance and differentiation of hESCs

Human ESCs (H9/WA09 line; WiCell) were cultured on plates coated with 10 μg/ml vitronectin (Stem Cell Technologies) in 37°C and 5% CO_2_. For maintenance, cells were daily supplied with E6 media [32] supplemented with 2 ng/mL transforming growth factor b (TGFb; Bio-Techne) and 25 ng/mL fibroblast growth factor 2 (FGF2; Dr. Marko Hyvönen, Cambridge University), and were passaged every 5–7 days using 0.5 mM EDTA (Thermo Fisher Scientific) in phosphate-buffered saline (PBS, Thermo Fisher Scientific). TeSR medium (Stem Cell Technologies) was instead used before and after the nucleofection (detailed below). Antibiotics were not used. For two-dimensional directed differentiation, pluripotent hESCs were plated as single cells at 4.0–5.0x10^4^ cells/cm^2^ using Accutase (Gibco) and 10 μM Y27632 (Selleck). After two days in the maintenance media, cells were supplied with the differentiation media every 24 hour as described in [33] with slight modifications. END day 1: CDM-PVA media supplemented with 80 ng/ml FGF2, 10 μM LY294002 (Promega), 10 ng/ml bone morphogenetic protein 4 (BMP4; Bio-Techne), 100 ng/ml Activin A (Dr. Marko Hyvönen, Cambridge University) and 3 μM CHIR99021 (Tocris Bioscience). END day 2: CDM-PVA media supplemented with 80 ng/ml FGF2, 10 μM LY294002, 10 ng/ml BMP4 and 100 ng/ml Activin A. END day 3: RPMI-B27 media supplemented with 80 ng/ml FGF2 and 100 ng/ml Activin A. END day 4–8: RPMI-B27 media supplemented with 50ng/ml Activin A. NEU day 1–2: CDM/PVA media supplemented with 20 ng/ml FGF2, 3 μM CHIR99021, 0.1 μM LDN193189 (Sigma-Aldrich), 10 μM SB431542 (Tocris Bioscience). NEU day 3–6: N2B27 media supplemented with 10 μM SB431542.

### Quantitative RT-PCR

Total RNA was extracted from cells using the GenElute Mammalian Total RNA Miniprep Kit (Sigma-Aldrich) and the On-Column DNase I Digestion set (Sigma-Aldrich). Complementary DNA was synthesized from the RNA using random primers (Promega), dNTPs (Promega), RNAseOUT (Invitrogen) and SuperScript II (Invitrogen). Real-time PCR was performed with KAPA SYBR FAST qPCR Master Mix (Kapa Biosystems) on QuantStudio 12K Flex and QuantStudio 5 Real-Time PCR System machines (Thermo Fisher Scientific). Molecular grade water (Thermo Fisher Scientific) was used when necessary. Each gene expression level was normalized by the average expression level of *PBGD*. Primer sequences are shown in S1 Table.

### Western blotting

Cells were washed once, scraped and spun down in a tube at 800 G for 3 min with PBS. Pellets were lysed in CelLytic Medium (Sigma-Aldrich) containing cOmplete, EDTA-free Protease Inhibitor Cocktail (Merck) and PhoSTOP phosphatase inhibitor (Sigma-Aldrich) for 15 min at room temperature with gentle mixing. Lysates were centrifuged at 17 KG for 5 min at 4°C, and supernatants were applied for Pierce BCA Protein Assay Kit (Thermo Fisher Scientific) according to the manufacturer’s instructions in order to quantify the total protein content. Equal amounts of proteins among samples were denatured at 90ºC for 5 min with 1x NuPAGE LDS Sample Buffer (Thermo Fisher Scientific) and 1% β-mercaptoethanol (Sigma-Aldrich), and were electrophoresed together with Precision Plus Protein Ladder (Bio-Rad) by XCell SureLock Mini-Cell (Thermo Fisher Scientific) using 4–12% NuPAGE Bis-Tris Precast Gels (Thermo Fisher Scientific) and NuPAGE MOPS SDS Running Buffer (Thermo Fisher Scientific). SDS-PAGE gels were then transferred to PVDF membranes (Bio-Rad) by Mini Trans-Blot Cell (Bio-Rad) using NuPAGE Transfer Buffer (Thermo Fisher Scientific) and Methanol (Thermo Fisher Scientific). Membranes were incubated with 0.1% Tween-20 (Sigma-Aldrich) in PBS (PBST) containing 4% skim milk (Marvel) for 30 min at room temperature, with primary antibodies overnight at 4ºC, and with secondary antibodies for 1 h at room temperature. The primary antibodies (RB: 554136, BD Life Sciences, 1:500; RBL1: sc-318, Santa Cruz, 1:500; RBL2: 610261, BD Life Sciences, 1:500; α-Tubulin: T9026, Sigma-Aldrich, 1:40,000) and HRP-conjugated secondary antibody (Mouse: A2554; Rabbit, A0545, both from Sigma Aldrich and at 1:10,000) were dissolved in 4% skim milk in PBST, and each staining was followed by three PBST washes. Finally, signals were detected using Pierce ECL Western Blotting Substrate (Thermo Fisher Scientific) and X-Ray Super RX Films (Fujifilm).

### Simultaneous knockout of RBL1 and RBL2 by CRISPR/Cas9 plasmids

Single guide RNA (gRNA) sequences targeting exon 2 of *RBL1* and exon 3 of *RBL2* were designed using the DESKGEN online designing tool and selected based on the predicted activity and off-target scores. Complementary oligo nucleotides were synthesized by Sigma-Aldrich, annealed to form gRNA seed sequences, and ligated into the pSpCas9(BB)-2A-Puro plasmid (PX459, Addgene) digested with BbsI (Thermo Fisher). Resultant vectors were checked by Sanger sequencing (GENEWIZ) and prepared for transfection by QIAquick Maxiprep Kit (Qiagen). Nucleofector device and kits (Lonza) were used to deliver the plasmids to the cells according to the manufacturer’s instructions (Solution: Primary Cell P3, Pulse Code: CA 137). Cells were dissociated into 3–4 cell clumps by Accutase and 2x10^6^ clumps were used for one shot of electroporation. 24 hours after the nucleofection, cells transiently expressing the plasmids were selected by 1 μg/ml puromycin within 24 hours. Recovered clonal colonies were picked up manually approximately 2 weeks after selection. Genome DNA was collected with QuickExtract DNA Extraction Solution (Epicentre), and was amplified at the target sites using TitaniumTaq (TaKaRa). PCR products were purified with Exosap-it reagent (Affymetrix) and sent for Sanger sequencing. TIDE algorithm (https://tide-calculator.nki.nl/) was used to identify wild types, heterogenous mutants and homogenous mutants. Each gRNA was transfected separately into wild type H9 cells to examine the targeting efficiencies, and then two best gRNAs for each RBL1 or RBL2 were selected and transfected in combinations. See also Fig 2A for the overall procedure and S1 Table for the sequences of gRNAs and genotyping primers.

### Inducible RB knockdown

Short hairpin RNA (shRNA) sequences against *RB* were selected from MISSION shRNA library provided by Sigma-Aldrich and the best efficient shRNA was used for the further experiments. Construction and transfection of sOPTiKD plasmids for RB and cloning of the transfectant cells were carried out as described in [20]. Nucleofection was used for transfection as described above. To induce knockdown, tetracycline (Sigma-Aldrich) dissolved in Embryo Transfer Water (Sigma-Aldrich) was added at 1 μg/ml in the maintenance or differentiation media. See S1 Table for the shRNA sequence.

### Immunocytochemistry

Cells plated on vitronectin-coated round coverslips (Scientific Laboratory Supplies) were washed once with PBS, and fixed with 4% paraformaldehyde (Alfa Aesar) in PBS. Following another PBS wash, cells were incubated with 0.25% Triton (Sigma-Aldrich) in PBS at 4°C for 15–20 min, 0.5% BSA (Sigma-Aldrich) in PBS at room temperature for 30 min, primary antibodies at 4°C overnight and secondary antibodies at room temperature for one hour. Primary antibodies (POU5F1: sc-5279, Santa Cruz; NANOG: ab21624, Abcam; SOX2: AF2018, R&D, reconstituted at 0.2 mg/ml in PBS) and secondary antibodies (anti-Mouse IgG: A31571; anti-Rabbit IgG: A10042; anti-Goat IgG: A11055, Invitrogen) together with 10 μg/ml Hoechst33258 (Sigma-Aldrich) were diluted in 0.5% BSA in PBS at [1:200] and [1:1000], respectively. Each staining was followed by three washes with 0.5% BSA in PBS. Coverslips were preserved on slide glasses (Corning) with ProLong Gold Antifade Mountant (Life Technologies) and nail polish, and observed with LSM 710 inverted confocal system (Zeiss).

### Cell cycle profile analysis

To quantify the cells in each cell cycle phase, Click-iT EdU Alexa Fluor 488 Flow Cytometry Assay Kit (C10420, Thermo Fisher) was used according to the manufacture’s instruction. In brief, cells were incubated with 25 μM 5-ethynyl-20-deoxyuridine (EdU) for one hour and collected using Cell Dissociation Buffer (Gibco) following a PBS wash. Cells were fixed with 4% paraformaldehyde for 15 min at room temperature and washed with 1% BSA in PBS. Cells were then permeabilized for 15 min with saponin-based permeabilization/wash buffer and incubated with the Click-iT reaction cocktail for 30 min protected from light. Cells were washed once with saponin-based permeabilization/wash buffer and stained for DNA content using the FxCycle Far Red dye (Invitrogen). Cells were analyzed with Fortessa cell analyzer (BD) and FlowJo software.

### RNA-seq analysis

Template mRNA was extracted as described in 2.2. Paired-end fragment library preparation, sequencing with Illumina HiSeq 4000 sequencing system and alignment to GRCh38 were carried out in Sanger Institute. Reads were processed by Samtools [34] and counted by Subread [35]. Differential expression analysis and GO enrichment analysis were performed with DESeq2 package [36] and topGO package [37], respectively. The raw data files are available at ArrayExpress under accession number E-MTAB-11109.

## Conclusion

We established an inducible knockdown system of RB in hESCs deficient for *RBL1* and *RBL2*. These cell lines are useful for the detailed molecular roles of *RB* family genes in human pluripotency and lineage commitments. They revealed that RBs regulate cell cycle but not pluripotency in undifferentiated hESCs, and that they are critical for the marker gene expression during the foregut endoderm formation.

## Supporting information

S1 Fig Transcript levels of representative genes in the pluripotent tWTA and dKOA cells measured by qRT-PCR. Box-plot elements are same as described in Fig 1. n = 7 from three different experiments. To determine statistical significance between CTRL and TET sample groups from the same time point, Student’s t-test and Mann-Whitney test were performed based on Shapiro-Wilk normality test. No mark: p-value ≥ 0.05, ****: p-value < 0.0001.
(JPG)

S2 Fig A, Relative expression levels of neural marker genes in neuroectoderm 6 day. NS: p-value ≥ 0.05, ****: p-value < 0.0001 (Student’s t-test after Shapiro-Wilk normality test). n = 7. B, Relative expression levels of representative genes during the endoderm differentiation in the RBL1/2_tWT+RB_iKD and RBL1/2_dKO+RB_iKD hESCs from gRNA set A. Box-plot elements are same as described in Fig 1. n = 7 from three different experiments. To determine statistical significance between CTRL and TET sample groups on the same time point, Student’s t-test and Mann-Whitney test were performed based on Shapiro-Wilk normality test. No mark: p-value ≥ 0.05, *: p-value < 0.05, **: p-value < 0.01, ***: p-value < 0.001, ****: p-value < 0.0001. C, S and G2/M phases of the tWT and dKO cells during the endoderm differentiation. NS: p-value ≥ 0.05 (Ordinary one-way ANOVA test after Shapiro-Wilk normality test). n = 3 or 5.
(JPG)

S3 Fig RNA-seq analyses of samples from endoderm day 3. A, Number of DEGs identified in each comparison (FC > 1). B, Gene ontology enrichment analysis on the DEGs detected between tWT CTRL and dKO TET (black box in A).
(JPG)

S1 Table Sequences of the oligonucleotides used in this work. (XLSX)

S1 Raw images (PDF)
