# Supplementary figures and images for: Simultaneous depletion of RB, RBL1 and RBL2 affects endoderm differentiation of human embryonic stem cells
Source: PLoS One. 2022 Nov 22;17(11):e0269122. doi: 10.1371/journal.pone.0269122 (PMC9681086; doi:10.1371/journal.pone.0269122)

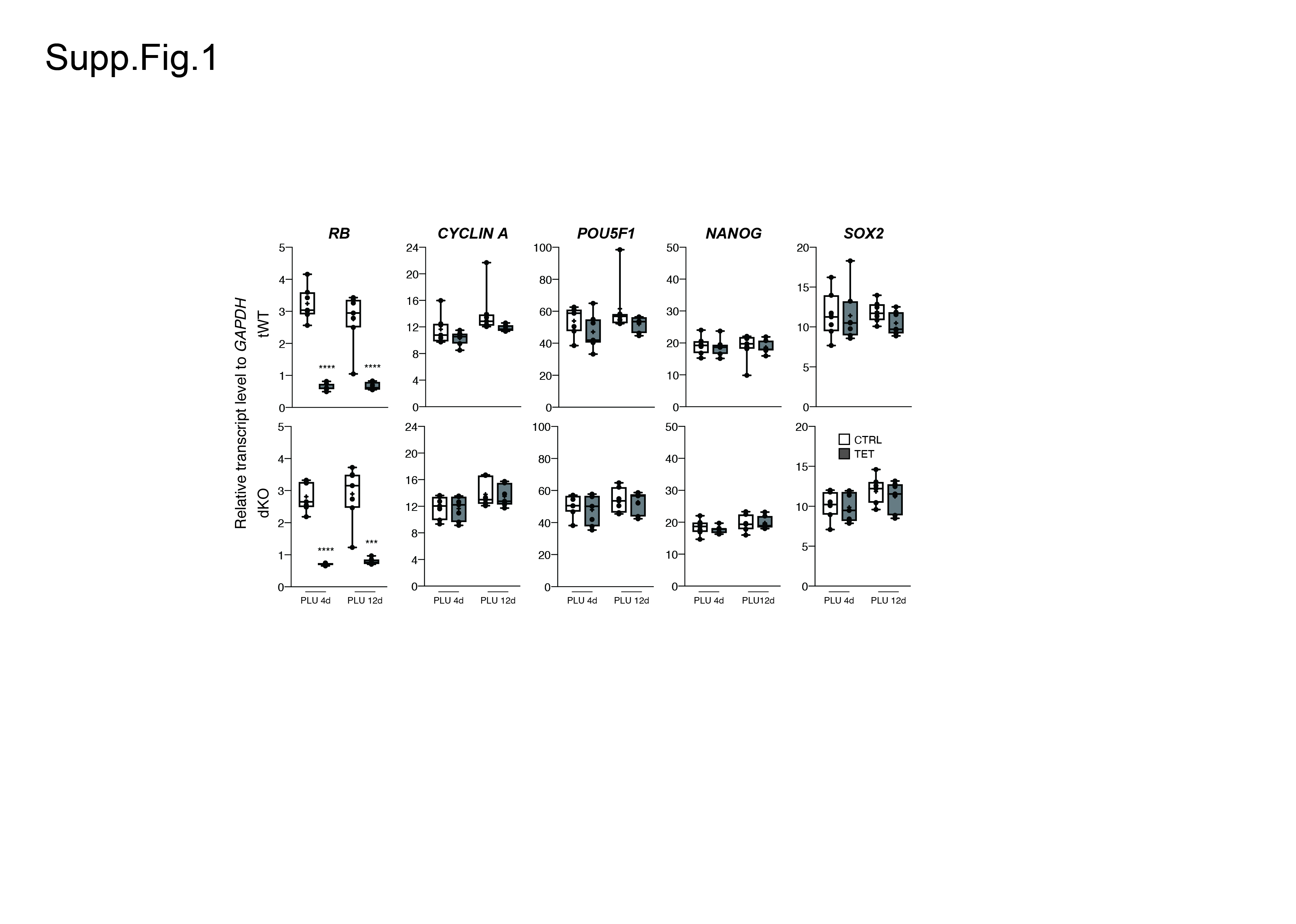

Supplement: S1 Fig — Box-plot elements are same as described in Fig 1. n = 7 from three different experiments. To determine statistical significance between CTRL and TET sample groups from the same time point, Student’s t-test and Mann-Whitney test were performed based on Shapiro-Wilk normality test. No mark: p-value ≥ 0.05, ****: p-value < 0.0001. (JPG) [file pone.0269122.s001.jpg]

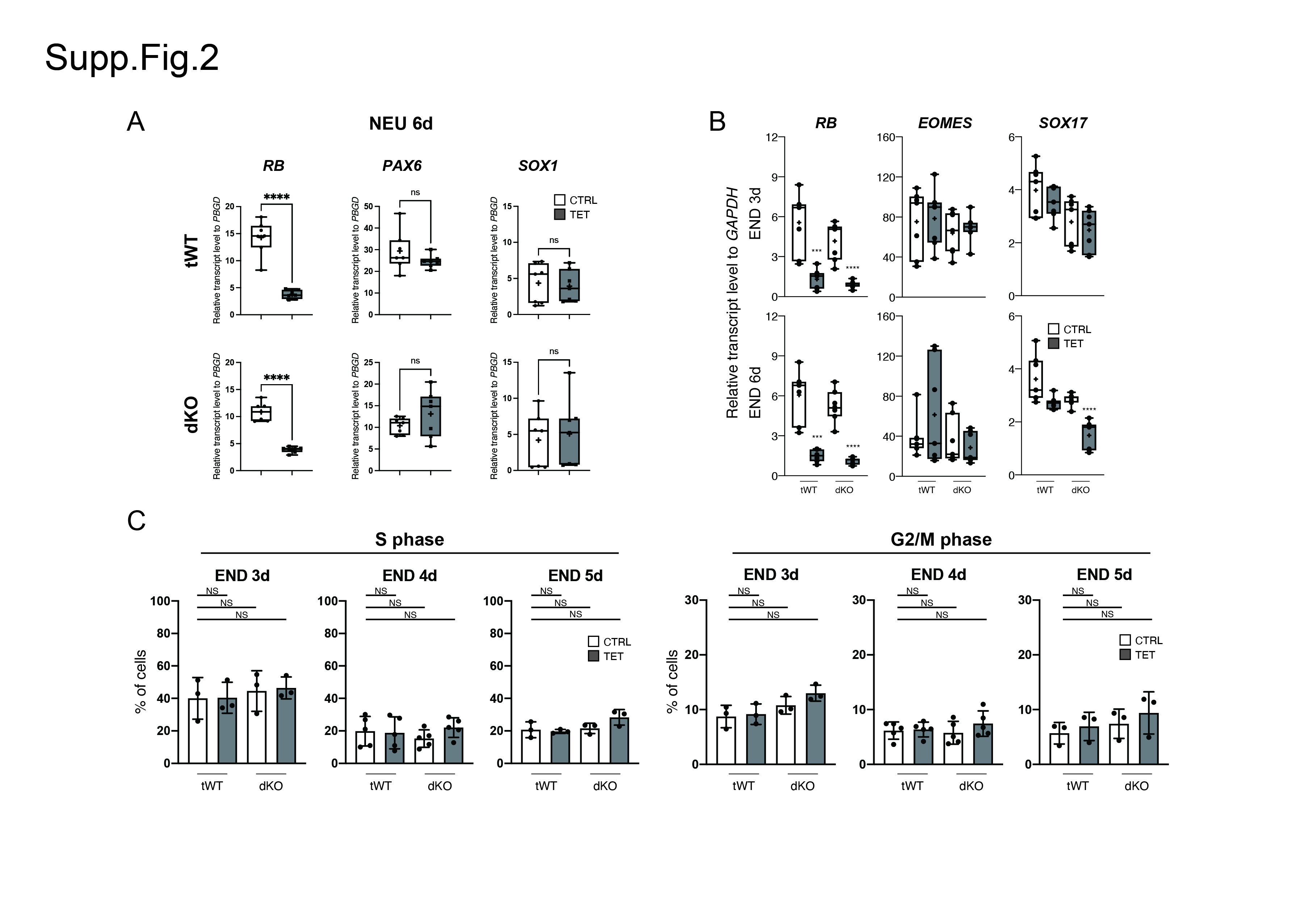

Supplement: S2 Fig — A, Relative expression levels of neural marker genes in neuroectoderm 6 day. NS: p-value ≥ 0.05, ****: p-value < 0.0001 (Student’s t-test after Shapiro-Wilk normality test). n = 7. B, Relative expression levels of representative genes during the endoderm differentiation in the RBL1/2_tWT+RB_iKD and RBL1/2_dKO+RB_iKD hESCs from gRNA set A. Box-plot elements are same as described in Fig 1. n = 7 from three different experiments. To determine statistical significance between CTRL and TET sample groups on the same time point, Student’s t-test and Mann-Whitney test were performed based on Shapiro-Wilk normality test. No mark: p-value ≥ 0.05, *: p-value < 0.05, **: p-value < 0.01, ***: p-value < 0.001, ****: p-value < 0.0001. C, S and G2/M phases of the tWT and dKO cells during the endoderm differentiation. NS: p-value ≥ 0.05 (Ordinary one-way ANOVA test after Shapiro-Wilk normality test). n = 3 or 5. (JPG) [file pone.0269122.s002.jpg]

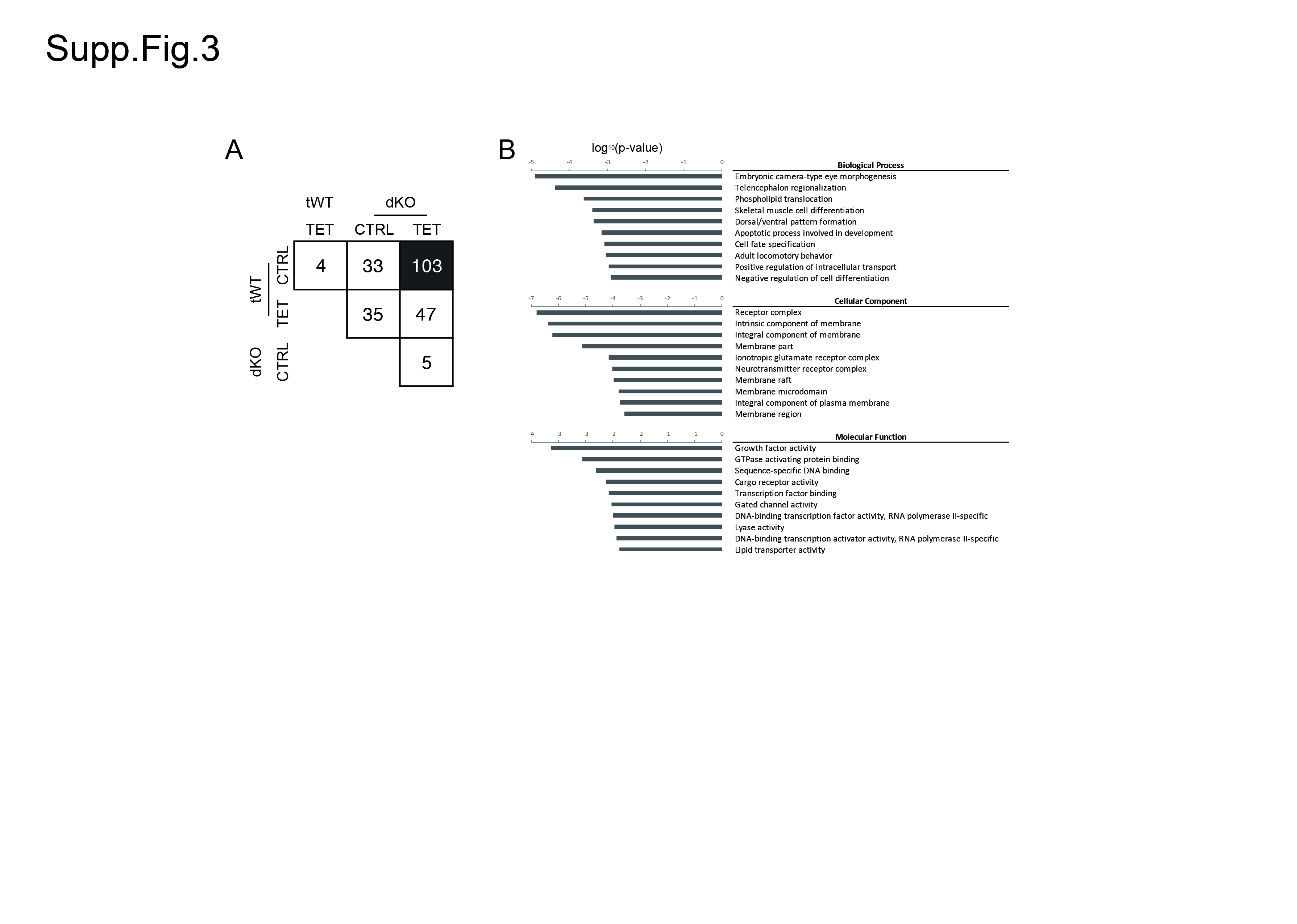

Supplement: S3 Fig — A, Number of DEGs identified in each comparison (FC > 1). B, Gene ontology enrichment analysis on the DEGs detected between tWT CTRL and dKO TET (black box in A). (JPG) [file pone.0269122.s003.jpg]

180831 15 min

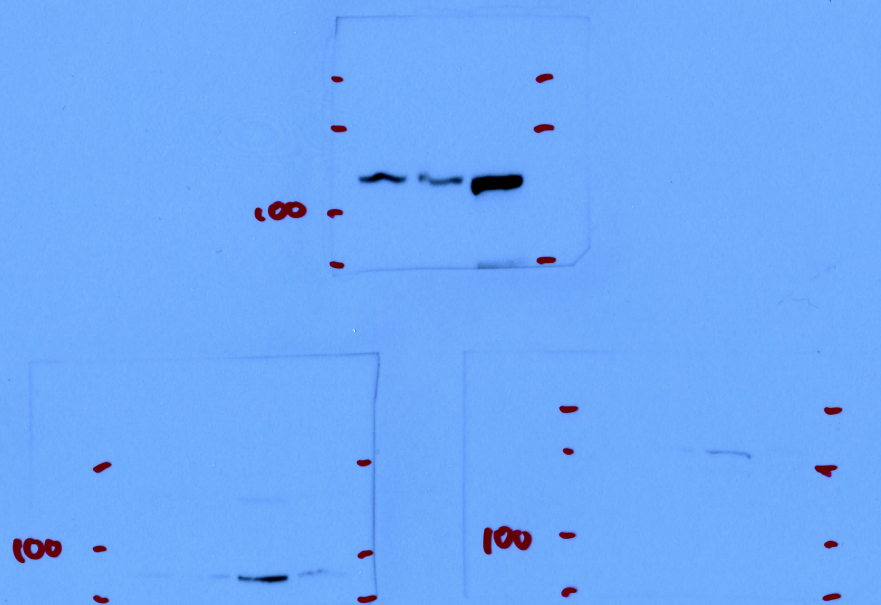

180631 60 min

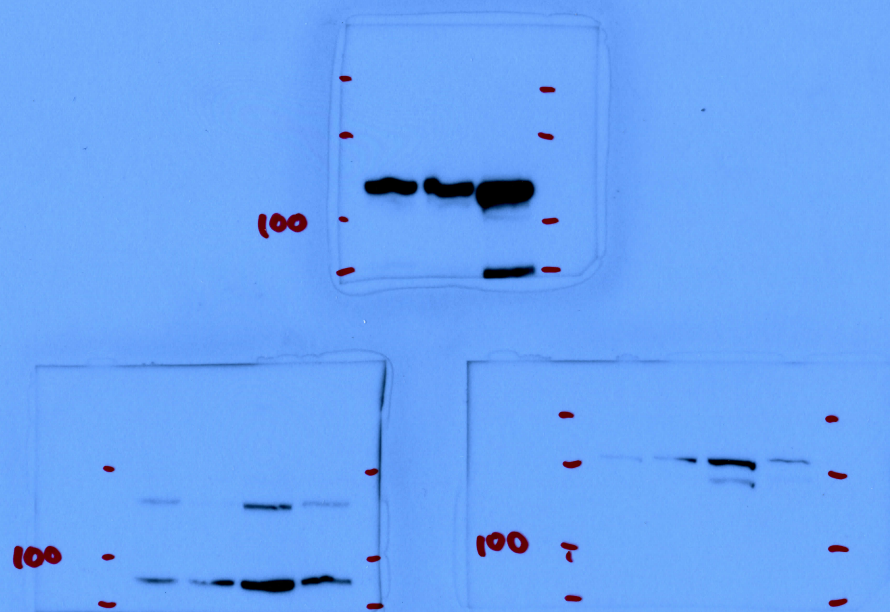

180831  $\alpha$ -TUB 15-20

180831  $\alpha$ -TUB

- - -

50 - - -

- - -

(80A07) 30 min

PBL 5278 [%100]

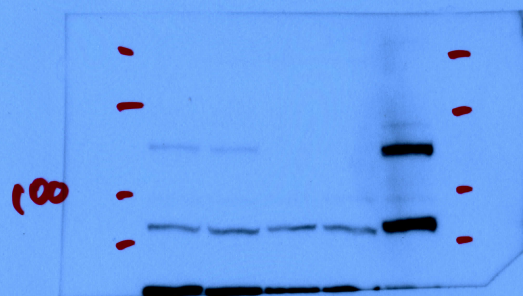

(80907 bovin

PBL1 (SC-318)

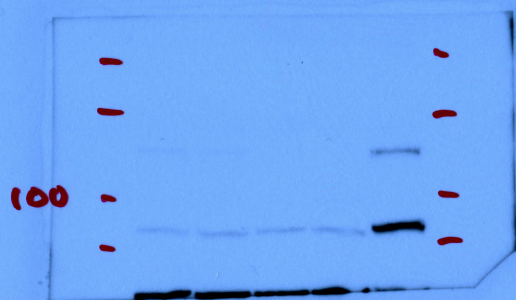

PBL2 (80610261)

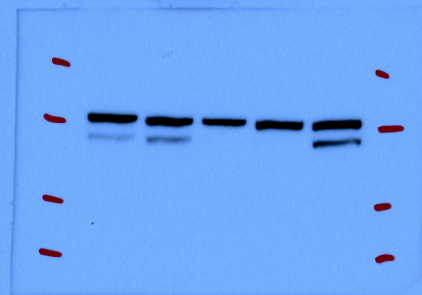

180907 10 sec  $\alpha$ -TUB

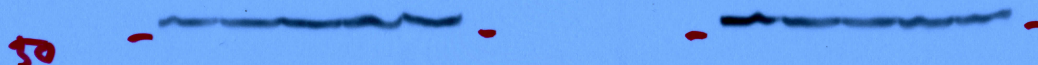

181016 PB bouin

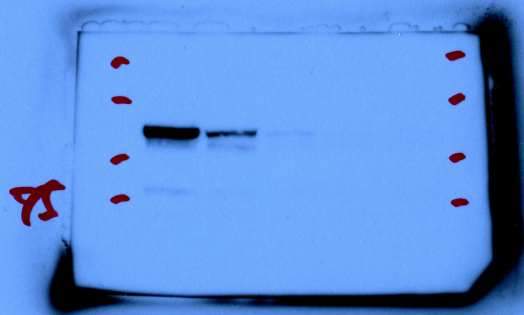

18016  $\alpha$ -Tub 2win

mem ①

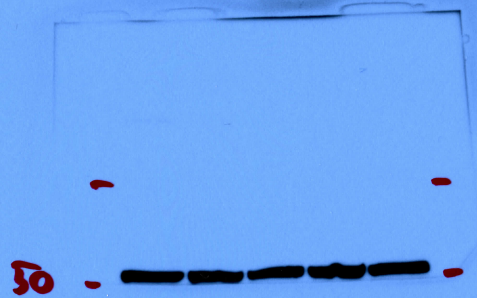

Supplement: S1 Raw images — (PDF) [file pone.0269122.s005.pdf]
